# Potentiated virucidal activity of pomegranate rind extract (PRE) and punicalagin against *Herpes simplex* virus (HSV) when co-administered with zinc (II) ions, and antiviral activity of PRE against HSV and aciclovir-resistant HSV

**DOI:** 10.1371/journal.pone.0179291

**Published:** 2017-06-30

**Authors:** David M. J. Houston, Joachim J. Bugert, Stephen P. Denyer, Charles M. Heard

**Affiliations:** 1 School of Pharmacy and Pharmaceutical Sciences, Cardiff University, Cardiff Wales, United Kingdom; 2 Department of Microbiology and Infectious Diseases, School of Medicine, Cardiff University, Cardiff Wales, United Kingdom; University of Cincinnati School of Medicine, UNITED STATES

## Abstract

**Background:**

There is a clinical need for new therapeutic products against *Herpes simplex* virus (HSV). The pomegranate, fruit of the tree *Punica granatum L*, has since ancient times been linked to activity against infection. This work probed the activity of pomegranate rind extract (PRE) and co-administered zinc (II) ions.

**Materials and methods:**

PRE was used in conjunction with zinc (II) salts to challenge HSV-1 and aciclovir-resistant HSV in terms of virucidal plaque assay reduction and antiviral activities in epithelial Vero host cells. Cytotoxicity was determined by the MTS assay using a commercial kit.

**Results:**

Zinc sulphate, zinc citrate, zinc stearate and zinc gluconate demonstrated similar potentiated virucidal activity with PRE against HSV-1 by up to 4-fold. A generally parabolic relationship was observed when HSV-1 was challenged with PRE and varying concentrations of ZnSO_4_, with a maximum potentiation factor of 5.5. Punicalagin had 8-fold greater virucidal activity than an equivalent mass of PRE. However, antiviral data showed that punicalagin had significantly lower antiviral activity compared to the activity of PRE (EC_50_ = 0.56 μg mL^-1^) a value comparable to aciclovir (EC_50_ = 0.18 μg mL^-1^); however, PRE also demonstrated potency against aciclovir-resistant HSV (EC_50_ = 0.02 μg mL^-1^), whereas aciclovir showed no activity. Antiviral action of PRE was not influenced by ZnSO_4_. No cytotoxicity was detected with any test solution.

**Conclusions:**

The potentiated virucidal activity of PRE by coadministered zinc (II) has potential as a multi-action novel topical therapeutic agent against HSV infections, such as coldsores.

## Introduction

The *Herpes simplex* virus is a member of the *Herpes viridae* family and infects a high proportion of the populace, causing a range of diseases from mild uncomplicated mucocutaneous (eg coldsores) to more serious infections, such as keratitis. HSV-1 is the causal agent of coldsores and encephalitis, while HSV-2 is normally associated with anogenital infections, which can transfer from mothers to neonates. The current gold standard treatment for HSV infections is aciclovir, a guanine analogue antiviral drug, which has a virustatic mode of action and is more effective in early-stage coldsores. Coupled with the emergence of *Herpes* strains that are resistant to nucleoside analogues, including aciclovir [1], there exists an urgent clinical need for new and more effective treatments for HSV infections, ideally ones that exert virucidal activity. This work concerns the development of such a new therapeutic system based upon the potentiated activity against HSV of pomegranate rind extract (PRE). For the purposes of this work the terminology; virucidal, pertains to the effect of a chemical or agent that prevent infection due to physical changes to the virus effectively ‘killing’ them and virustatic, pertaining to the interference of the viral life cycle and biological processes that preventing replication of the virus.

The pomegranate, fruit from the *Punica granatum L*. tree, is part of the punicaceae family native to the Middle East and has been used since antiquity as a remedy for a range of clinical conditions. The juice, seed, peel and rind of the pomegranate contain a wide range of phytochemicals [2] and there has been a substantial amount of research conducted upon different pomegranate components and chemical extracts. The major concentration of phytochemicals is within the pericarp and these include tannins, anthocyanidins such as delphinidin, cyanidin and pelargonidin, proanthocyananidins and various flavonoids, with significant antioxidant activity demonstrated in various *in vitro* models [3]; the antimicrobial effects of hydrolysable tannins was recently reviewed [4]. Antibacterial bioactivity of PRE has been observed using fractions extracted with acetone and methanol against both Gram-positive and Gram-negative bacteria. Complete growth inhibition of *B*. *cereus*, *B*. *subtilis*, *B*. *coagulens*, *S*. *aureus*, *E*. *coli* and *P*. *aeruginosa* was shown at 300 ppm [5]. The inhibition of α-glucosidase and human leukocyte elastase activity by PRE has been reported [6]—this action was claimed to impede viral attachment and penetration via disruption of the glycosylation of viral glycoproteins. Pomegranate juice extracts have antiviral effects against human immunodeficiency virus (HIV); the mechanism of action appeared to inhibit the binding of the HIV-1 envelope glycoprotein gp 120, preventing the interaction of HIV-1 with the CD4 receptor, and thus reducing the infectivity of the virus [7].

The phytochemicals of particular interest within PRE are the hydrolysable tannins: punicalin, gallic and ellagic acid esters of glucose and most notably punicalagin [8]. Punicalagin (MW 1084.7) and its major degradation product ellagic acid which are thought to be the major bioactive phytochemicals present in PRE [9]. Punicalagin is a large polyphenolic compound with a molecular mass of 1084.7 and is the most abundant tannin in PRE, constituting 80–85% w/w of the total. The structure consists of two subunits, punicalin and ellagic acid, linked via a hexose bridge and the overall molecule exists in one of 2 isomeric forms (anomers)–punicalagin α and β in a ratio of approximately 1:2 respectively. It was shown that punicalagin targeted and inactivated HSV-1 viral particles and could prevent binding (attachment), penetration, and cell-to-cell spread, as well as secondary infection [10]. Punicalagin was further reported to be effective in abrogating infection by human cytomegalovirus (HCMV), hepatitis C virus (HCV), dengue virus (DENV), measles virus (MV), and respiratory syncytial virus (RSV), at μM concentrations and in dose-dependent manners without significant cytotoxicity [11]; although the effect of punicalagin was not investigated as a potential virucide.

Antimicrobial or microbicidal potentiation can be described as significantly increased activity of an agent in the presence of a second, generally inactive agent. The scope for potentiated virucidal activity of PRE was revealed in the combination of PRE and ferrous sulphate (FeSO_4_) in a pH4.5 buffer [12]. The combination of PRE and FeSO_4_ was reported to produce a potent eleven-log reduction in the plaque forming ability of *Pseudomonas aeruginosa* and *Escherichia coli* phages [13] within two minutes of application of an aqueous mixture at room temperature [12]. Significant and detrimental damage was observed to the head and tail regions as well as to their overall integrity. Despite its demonstrable potency, the virucidal action of PRE and FeSO_4_ is fugitive and rapidly diminishes once the co-administration has taken place; which coincides with a blackening of the solution and has been attributed to oxidation of the ferrous ion to ferric ion, possibly involving a Fenton reduction mechanism [14]. Consequently our attention turned to examining the effect of zinc (Zn II) as an alternative potentiating/synergizing agent as it is relatively resistant to further changes in oxidation state, is colourless and has been reported as possessing innate virucidal activity [15, 16] including against HSV [17, 18]. In the current work we examined both the potentiated virucidal and anti-viral activities of simultaneous PRE and Zn (II) or punicalagin and Zn (II) challenge against HSV-1 and aciclovir-resistant HSV; cytotoxicity was determined using a commercial test kit.

## Materials and methods

### Materials

Pomegranates, obtained from a local supermarket, were of Spanish origin. Punicalagin (≥98%), ellagic acid (≥95%), zinc gluconate, zinc citrate, zinc oxide, zinc iodide, zinc nitrate and zinc stearate were purchased from Sigma-Aldrich, Gillingham, UK. Zinc sulphate (ZnSO_4_), Dulbecco's Modified Eagle Medium (DMEM), crystal violet, potassium hydrogen phthalate were purchased from Fisher Scientific (Loughborough, UK). HSV-1 17+ (NCPV00618), HSV-2 HG52 (NCPV00151), HSV-2 132349 ACV-res (NCPV00183) and Vero (green monkey kidney epithelial) cells (ATCC-CCL81) were obtained from the European Collection of Authenticated Cell Cultures (ECACC) (Salisbury, UK). The CellTiter 96 AQueous One Solution Cell Proliferation Assay (MTS) kit was obtained from Promega, Southampton, UK.

### Preparation of pomegranate rind extract (PRE)

Six fresh pomegranates were peeled and the rinds cut into thin strips approximately 2 cm in length then blended in deionised H_2_O (25% w/v) before being boiled for approximately 10 min. The crude suspension was then centrifuged at 10,400 x g at 4°C for 30 min using a Beckman Coulter Avanti J25 Ultracentrifuge, before being vacuum filtered through a 0.45μm nylon membrane filter (Merck Millipore Ltd, Cork, Ireland). The solution was then freeze-dried, occluded from light and stored at -20°C until required. The PRE was reconstituted by adding 2 mg to 10 mL of pH 4.5 phthalate buffer and sonicated for 10 min at 50–60 Hz and, once fully dissolved, sterilized by filtering through a 0.45μm Millex-FG syringe driven filter. The punicalagin concentration was analysed by HPLC and comprised 20% w/w of the freeze-dried material.

### Herpes virus stock generation

Viral stocks were grown in Vero cells (ATCC-CCL81). A T75 of vero cells was infected with the respective herpes virus from a titered stock at a multiplicity of infection of 0.01 and incubated until 50% of cells were losing adherence in the bottle, usually at day 3–4 post-infection. Cells and supernatant were collected, cells removed by low speed centrifugation, and the cell free supernatant stored at -70°C in aliquots of 1 mL. The titer of the stock was determined by plaque assay.

### Cell culture and virucidal plaque reduction assay

Vero cells were cultivated in DMEM and incubated at 37°C under 5% CO_2_ in an incubator. Cells were seeded at 5 x 10^5^ cells per well in a 24 well plate, 24 h prior to plaque assay. Virucidal activity was determined as log reduction. A virus suspension (10^6^ pfu/mL) was incubated with candidate virucidal test substance (30 min), prior to endpoint dilution (eg tenfold) and infection of a cell culture monolayer in a number of different wells of the same size to establish the titer. This was performed using a 24-well plate (24 WP) and each virion resulted in a localized area of infection known as a plaque, from which the number of plaque forming units (pfu) was quantified following crystal violet staining. Table 1 summarises the concentrations and combinations representing the challenges in the plaque reduction assay. Obtaining a direct comparison with punicalagin proved problematic due to the extremely low aqueous solubility of ellagic acid, therefore an alternative solvent was needed in which both species were soluble, and brief examination experiments showed that 10% dimethyl sulfoxide (DMSO) in deionised H_2_O was suitable.

**Table 1:** Zinc salt/tannin combinations used in virucidal assays.

Zinc compound	Tannin
Zinc oxide <0.00001M	± PRE 0.05 mg mL^-1^
Zinc nitrate 0.0005M	± PRE 0.05 mg mL^-1^
Zinc citrate 0.0005M	± PRE 0.05 mg mL^-1^
Zinc iodide 0.0005M	± PRE 0.05 mg mL^-1^
Zinc stearate 0.0005M	± PRE 0.05 mg mL^-1^
Zinc gluconate 0.0005M	± PRE 0.05 mg mL^-1^
ZnSO_4_ 0.5M (143.77 mg mL^-1^)	± PRE 0.05 mg mL^-1^ or Punicalagin 0.01 mg mL^-1^
0.1M (28.75 mg mL^-1^)	
0.05M (14.37 mg mL^-1^)	
0.01M (2.88 mg mL^-1^)	
0.005M (1.44 mg mL^-1^)	
0.001M (0.29 mg mL^-1^)	
0.0005M (0.14 mg mL^-1^)	
No addition	PRE 0.05 and 0.01 mg mL^-1^
No addition	Punicalagin 0.05 and 0.01 mg mL^-1^ (46.13 and 9.23 μM)
No addition	Ellagic acid 0.3 mg mL^-1^ (1 mM)

### Antiviral plaque assay

Most topical treatments for skin viral infections are based upon an antiviral mechanism, where the active compound inhibits viral replication. A plaque assay was used to assess the antiviral activity of the test compounds by quantifying infectious viruses. Antiviral activity was determined by the application of the test antiviral compound (pretreatment)/ challenge and then virus (after an incubation period) to confluent Vero cells in a 24WP. Preincubation of cells with the test compound followed by addition of the virus without rinsing away the compound remaining in the media, would be reflective of an in situ scenario within the skin. Each pure test substance was analysed in 5 different concentrations i.e. 100 μM, 50 μM, 10 μM, 5 μM and 1 μM and a control, with each dilution replicated in 4 wells; for PRE the concentrations were given in μg mL^-1^ as it has no definable molecular weight. Medium was removed under vacuum from the first row of four cells and 400 μL of test substance was applied. This was repeated until all medium was removed and replaced. Once the last well was filled the time was noted, and the plates incubated for 30 min to allow for any cellular absorption or reaction. After the 30 min incubation period, virus suspension (average 31.09 ± 3.09 pfu/well (range 28–34, n = 6)) was added to each well, and the 24 WP rocked/swirled to ensure an even spread of the virus. The plate was then incubated at 37°C for 45 min with rocking/swirling every 5 min, to ensure potential viral entry into the cells. Next, 300 μL of 1.2% Avicel overlay (Sigma-Aldrich, Gillingham, UK) was applied to each well in order to prevent infection of the monolayer by newly replicated virus released into the medium. The plate was incubated for three days, then stained using crystal violet. The test substances used with this method were PRE, ZnSO_4_, their combination, punicalagin, ellagic acid, phthalate buffer, alongside the established topical antiviral drug, aciclovir. The antiviral studies did not test above 100 μM for any compound. The EC_50_ was calculated from the sigmoid curve. Graphpad Prism 5 (GraphPad Software Inc, California, USA) was used to analyse the data.

### Cytotoxicity

Test solutions were analysed for cytotoxicity using the Cell Titer 96 AQueous assay method (Promega, Southampton, UK) and were performed in a 96 well plate (96WP) with a known concentration of Vero cells in each well. A density of 2.5 x 10^3^ cells per well were used for each plate prepared, by the trypsinisation of 80% confluent Vero cells in a T75 flask (Greiner Bio-One, Stonehouse, UK) and reconstituted in fresh DMEM Cells were seeded into a 96WP and incubated for 24 h at 37°C under 5% CO_2_. The medium was removed by aspiration, the cells washed three times with phosphate-buffered saline then immediately replaced with the dilutions of test materials. Cells were incubated for 6, 24, 48 and 72 h. Cell Titer 96 AQueous solution was used to detect cell viability and was added to each well and the plates incubated for 1 h at 37°C under 5% CO_2_. Optical density was then determined at 492 nm, with the blank subtracted from each sample reading and the density mean for the control cells arbitrarily assigned a value of 100% (n = 3).

## Results and discussion

### Effect of different Zinc (II) compounds on virucidal activity of PRE

**Fig 1:**
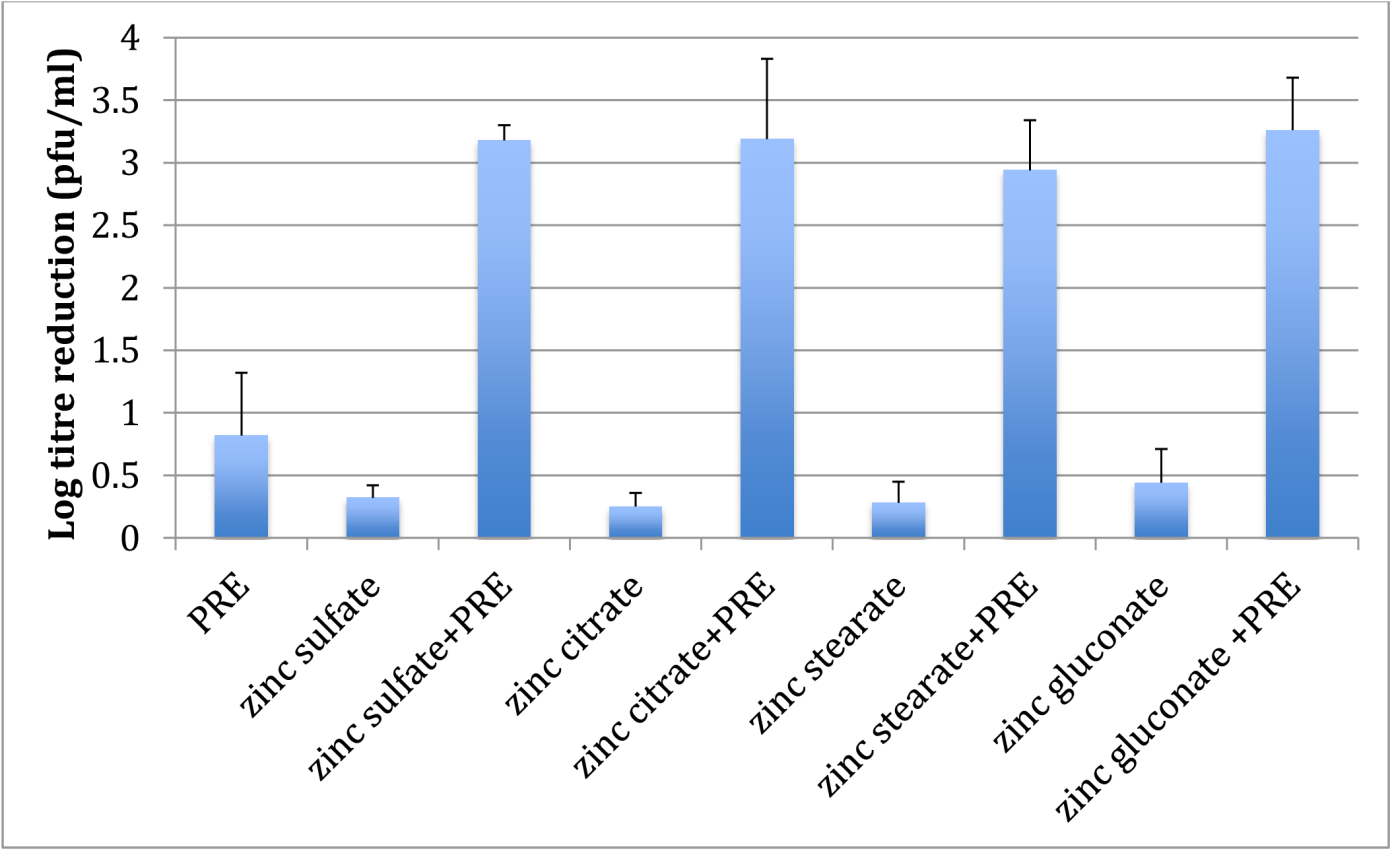
Virucidal log reduction data for the addition of PRE (0.05 mg mL^-1^) alone and when co-administered with a range of Zn (II) salts (0.5 mM) to HSV-1 (n = 3 ± SD). Results show the same potentiated activity for zinc sulphate, zinc citrate zinc stearate and zinc gluconate (p<0.05); thus confirming that zinc ions were responsible for the observed potentiated virucidal activity. However, zinc nitrate and zinc iodide both showed toxicity to the Vero host cells, whereas zinc oxide had limited solubility.

Fig 1 shows a plot of log reduction for PRE, a range of zinc salts and the combination. It can be seen that the salts alone provided very low/negligible log reductions, approx. 0.4, and across the range there was no statistically significant difference (p > 0.05). PRE alone demonstrated low activity with a log reduction of 0.82; however, when the PRE and zinc salt was coadministered, statistically significant (p<0.05) potentiation of the PRE was observed using zinc sulphate (3.88), zinc citrate (3.89) zinc stearate (3.59) and zinc gluconate (3.98). Although some variation was apparent, the differences were not statistically significant within this group of four salts (p > 0.05). In the case of zinc oxide, the solubility was very low and could only be evaluated at 10^−5^ M, rather than 10^−4^ M as for the other zinc salts. Furthermore, zinc oxide is amphoteric in nature and therefore not truly ionic, accounting for its very low aqueous solubility [19]. Virucidal activity of zinc nitrate and zinc iodide were not shown due to the high cytotoxicity of the mixture to the host Vero cells limiting the analysis at these concentrations; zinc iodide also proved to be cytotoxic to the host Vero cells [20]. This was apparent visually during the experiment by the full lysis of Vero cells and therefore lack of crystal violet staining. In a similar manner, it can be concluded that the nitrate ion was also cytotoxic to the Vero cells. However, the remaining salts were water-soluble (≥ 0.0005 M) and did not result in cell cytotoxicity. There were no obvious colour changes with any of the zinc salts upon the addition of PRE, although zinc iodide was intrinsically a brown-coloured solution and remained so throughout the experiment. These observations supported the chemical analyses carried out demonstrating the absence of any evidence for a redox reaction involving Zn^2+^ [21].

### Virucidal activity of PRE when combined with different levels of ZnSO_4_

**Fig 2:**
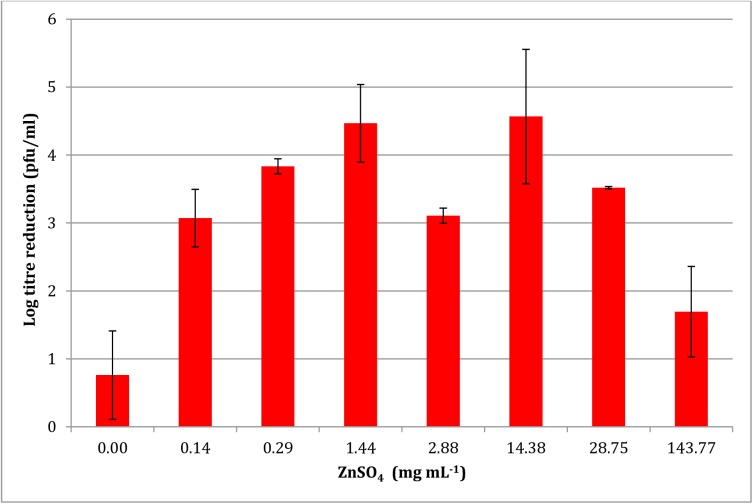
Effect of ZnSO_4_ concentration on the potentiated virucidal activity of PRE (0.05 mg mL^-1^) (n = 4 ± SD). Results show an increase in potentiated activity up to 1.44 mg mL^-1^; thereafter the pattern is unpredictable, and generally decreases with higher zinc levels.

Fig 2 shows the log reduction of HSV-1 with increasing levels of ZnSO_4_. A parabolic relationship was observed with maximal log reduction of 4.47 ± 0.11 and 4.56 ± 0.11 after the addition of 1.44 and 14.38 mg mL^-1^ of ZnSO_4_. After this point a decrease in virucidal activity was observed. It can also be seen that between the two maxima a statistically significant decrease in virucidal activity was observed at a ZnSO_4_ concentration of 2.88 mg mL^-1^ with a log reduction of 3.11 ± 0.57.

### Virucidal activity of equal masses of punicalagin and PRE with ZnSO_4_

**Fig 3:**
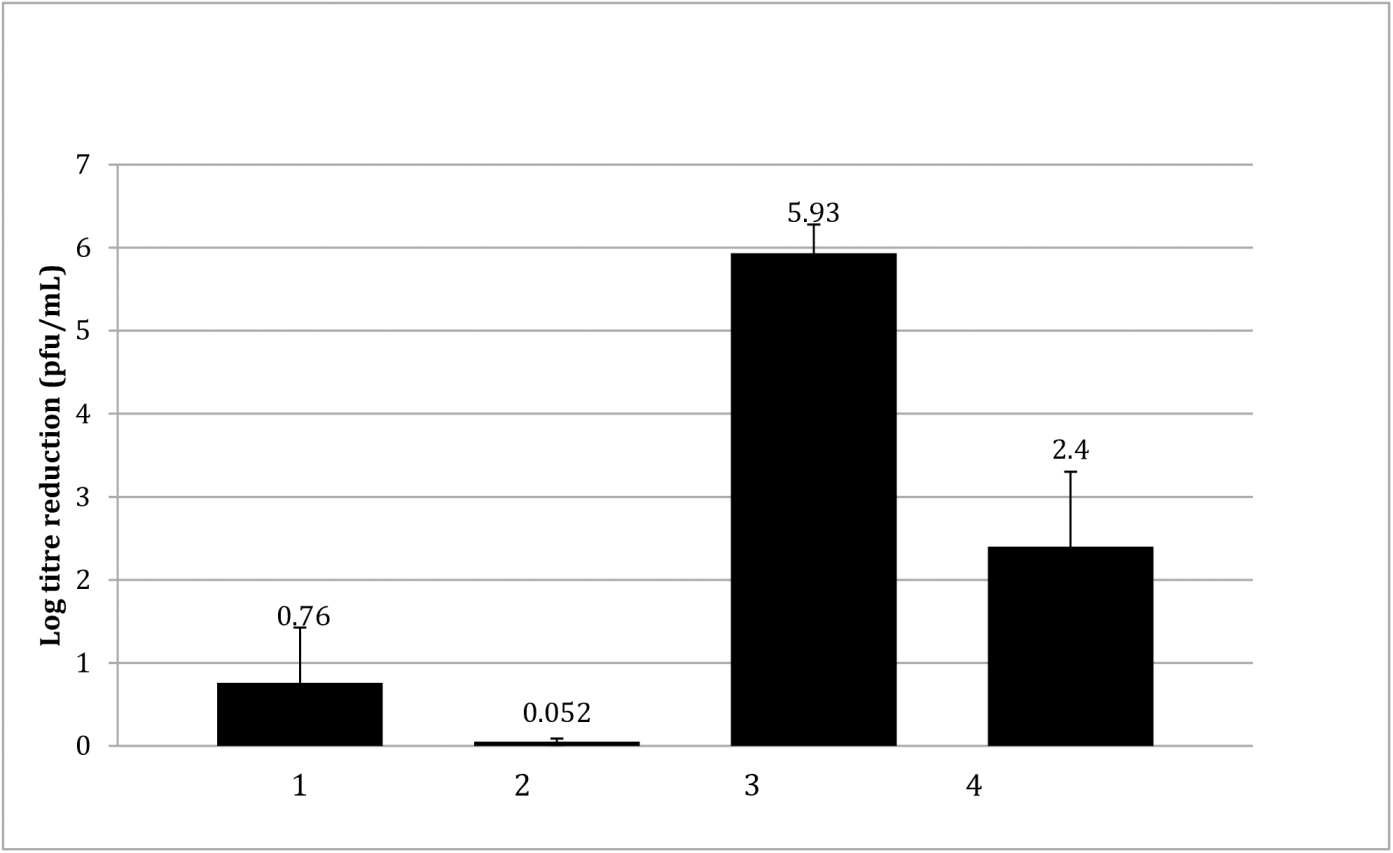
Comparison of the virucidal log reduction of HSV-1 by PRE and punicalagin against HSV-1 on a mass-to-mass basis (n = 4 ± SD). 1: PRE 0.05 mg mL^-1^; 2. PRE 0.01 mg mL^-1^; 3. punicalagin 0.05 mg mL^-1^ (46.1 μM); 4. punicalagin 0.01 mg mL^-1^ (9.22 μM). Results show greater potency achieved with equivalent concentrations of punicalagin compared to PRE.

The major phytochemical present in PRE is the ellagitannin, punicalagin [22], at approximately 20%, which has been reported as possessing microbicidal activity [23]. Here the effect of this compound on HSV was determined in isolation from the other components of PRE. The virucidal log reduction effected by punicalagin in comparison to PRE at 0.01 and 0.05 mg mL^-1^ is shown in Fig 3. The results show that the log reduction of HSV-1 pfu due to the isolated punicalagin (0.05 mg mL^-1^) was 5.93 ± 0.35, significantly greater (x7.8) than the log reduction due to PRE (0.05 mg mL^-1^) of 0.76 ± 0.66 (p < 0.05). It is important to note that a log reduction of 5.93 is the maximal limit for the test due to the viral titer used; however, the marked difference between the activities of PRE and punicalagin was again observed at the lower concentration of 0.01 mg ml^-1^ where the differential was even greater (x46.0).

A punicalagin level 0.01 mg mL^-1^ is representative of the concentration of this tannin within 0.05 mg mL^-1^ PRE (i.e. punicalagin accounts for approximately 20% of the dry mass of PRE, as determined by HPLC). By decreasing the concentration, the log reduction data of HSV-1 in Fig 3 show that the virucidal log reduction due to PRE decreased from 0.76 ± 0.66 to 0.05, and that punicalagin decreased from 5.93 to 2.4 ± 0.9. These results demonstrate the greatly enhanced virucidal activity of punicalagin on a mass-to-mass basis relative to PRE. The virucidal activity of PRE, if it is to be mainly attributed to the punicalagin content with the extract, appears partially compromised by other components of the extract.

### Virucidal activity of PRE and ellagic acid in isolation

**Fig 4:**
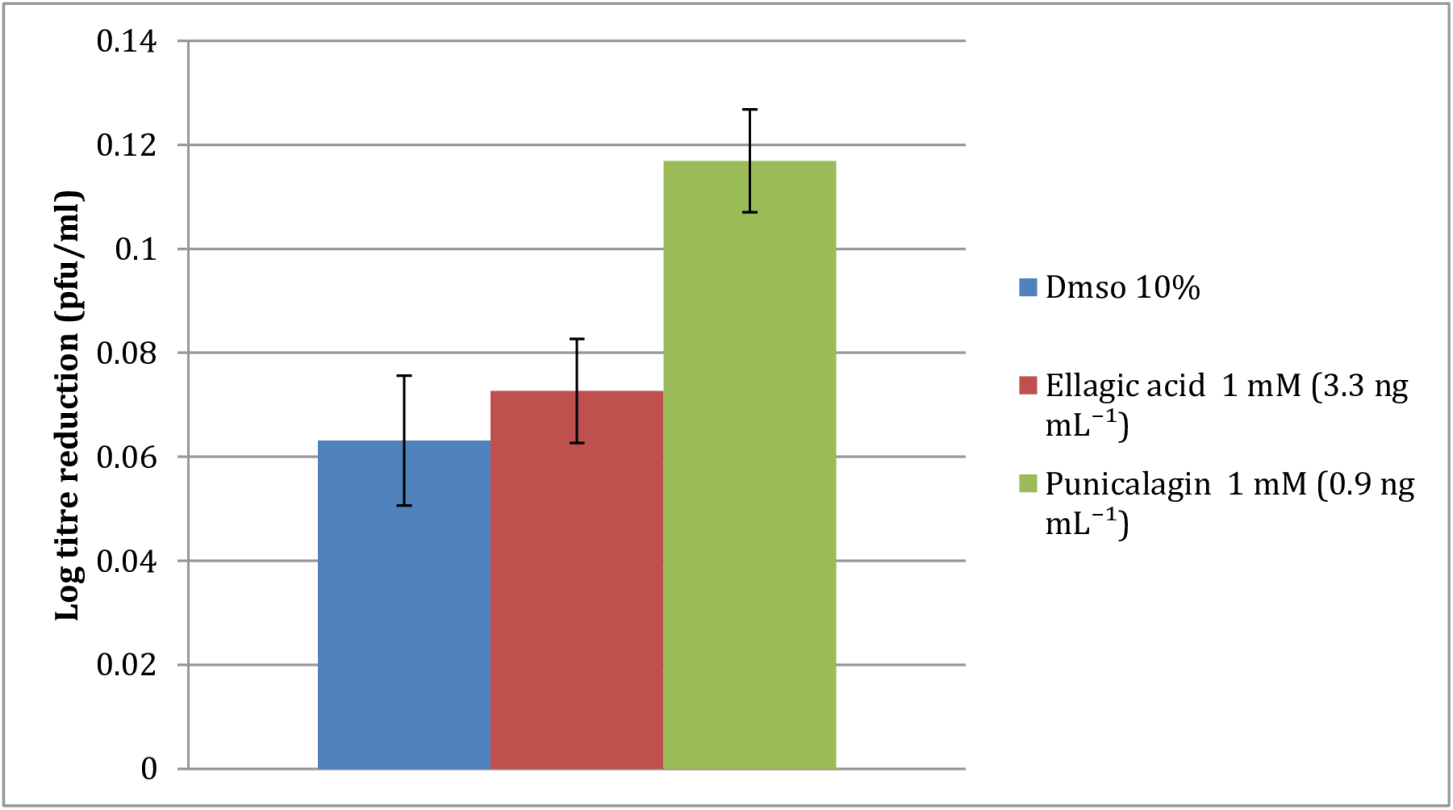
Virucidal log reduction due to the main punicalagin versus ellagic acid solubilised in 10% aqueous DMSO (n = 4 ± SD). Results show no activity of ellagic acid, whereas punicalagin was significantly greater than control.

Punicalagin, although in greatest proportion, is by no means the only phytochemical constituent of PRE. Here, we examined the effect of another ellagitannin, ellagic acid (EA), a direct breakdown product of the esterification of punicalagin. Fig 4 shows that the virucidal activity of EA 10% DMSO in deionised H_2_O was no different from blank solution (p >0.05); however, punicalagin showed significantly greater virucidal action than either the control or ellagic acid (p < 0.05).

### Virucidal activity of punicalagin in combination with ZnSO_4_

**Fig 5:**
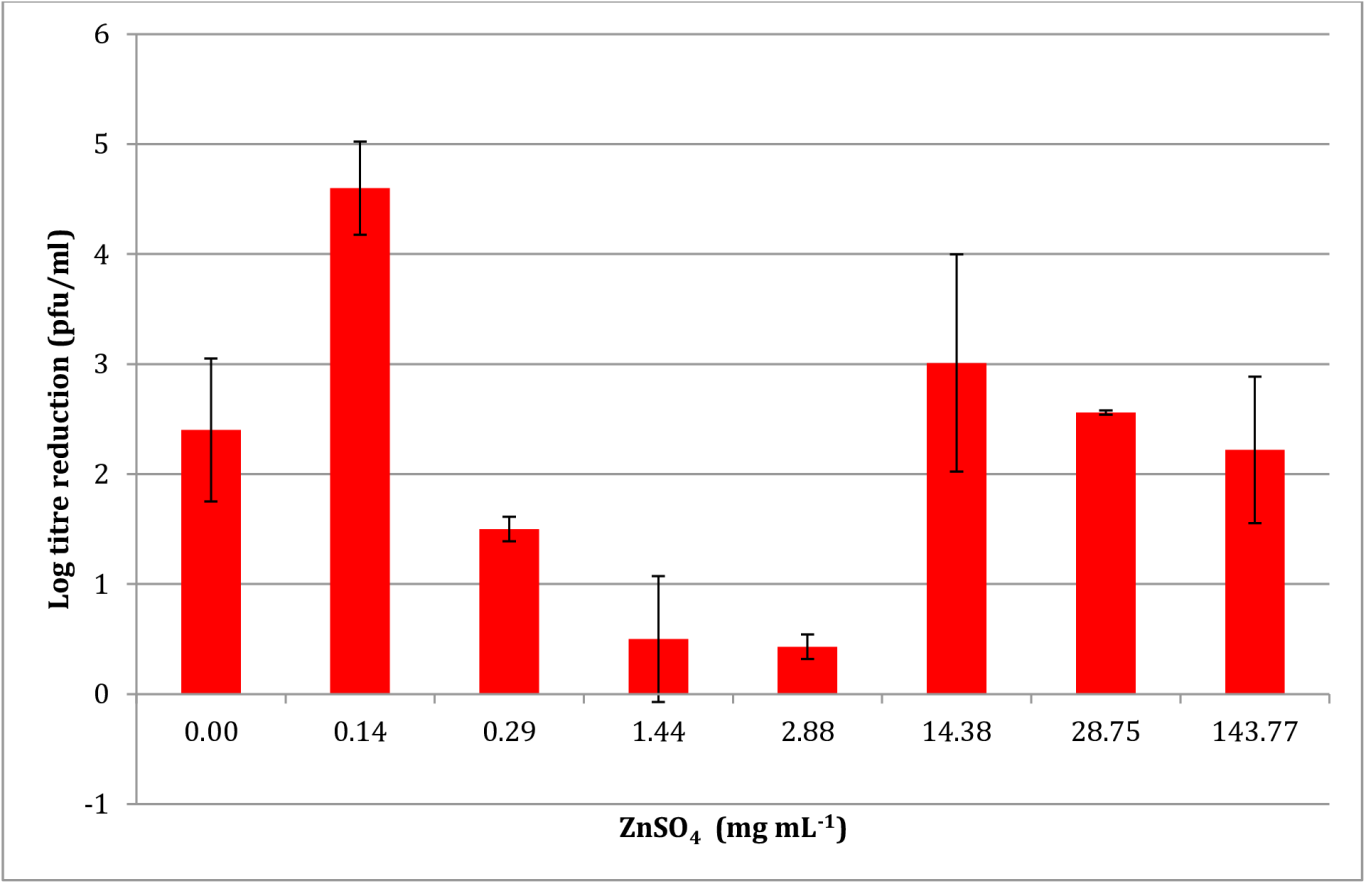
Effect of ZnSO_4_ concentrations on the potentiated virucidal activity of punicalagin (0.01 mg mL^-1^) (n = 4 ± SD). Results show a maximum at 1.44 mg mL^-1^, after which the pattern is unpredictable.

Fig 5 shows the potentiation by ZnSO_4_ of varying concentrations on the virucidal activity of 0.01 mg mL^-1^ punicalagin. As previously shown in Fig 4 punicalagin had innate virucidal effect resulting in a log reduction of 2.4 ± 0.9 (note: 0.01 mg mL^-1^ used here rather than 0.05 mg mL^-1^). The addition of ZnSO_4_ (0.14 mg mL^-1^) caused a large increase in potentiated virucidal action to 4.6 ± 0.16. However, as seen in the PRE experiment (Fig 3), the addition of further amounts of ZnSO_4_ caused a decrease in the virucidal activity below that of punicalagin alone. A biphasic trend was again observed, as at the higher concentrations of ZnSO_4_ (14.38, 28.75 and 143.77 mg mL^-1^) higher log reductions of 3.01, 2.56 and 2.22 were observed.

### Antiviral activity of PRE, punicalagin, ellagic acid and aciclovir against HSV-1 and aciclovir-resistant HSV-2

In this section, the viral replication inhibitory activities of PRE, punicalagin and ellagic acid were determined alongside the established topical antiviral drug, aciclovir. Two strains of *Herpes simplex* virus were used: HSV-1 and HSV-2 aciclovir resistant (ACR). Table 2 shows that the EC_50_ of aciclovir against HSV-1 was 0.81 μM (which is equivalent to 0.182 μg mL^-1^) and in general agreement with levels stated in the literature [24,25].

**Table 2:** The antiviral IC_50_ against *Herpes simplex* virus-1 (HSV-1) and acyclovir-resistant *Herpes simplex* virus (HSV-ACR) of PRE, punicalagin, ZnSO_4_ and ellagic acid in comparison to aciclovir (n = 3 ± SD). Note: IC_50_ concentration of PRE is in units of mass mL^-1^ rather than μM as it is a complex mixture of compounds.

Challenge	MW	Virus	IC_50_ (μg mL^-1^)	IC_50_ (μM)
**PRE + ZnSO_4_**	N/A	HSV-1	0.550 ± 0.062	N/A
**PRE**	N/A	HSV-1	0.560 ± 0.039	N/A
		HSV-ACR	0.016 ± 0.009	N/A
**Punicalagin**	1084.7	HSV-1	≥108.47	≥100
		HSV-ACR	≥108.47	≥100
**Ellagic acid**	302.2	HSV-1	≥30.22	≥100
		HSV-ACR	≥30.22	≥100
**ZnSO_4_**	161.47	HSV-1	≥16.15	≥100
		HSV-ACR	≥16.15	≥100
**Aciclovir**	225.21	HSV-1	0.182	0.81 ± 0.03
		HSV-ACR	≥22.52	≥100

The PRE extract exhibited potent antiviral properties against HSV-1 0.556 ± 0.39 μg mL^-1^ (concentration range 1, 0.5, 0.01, 0.005 and 0.001 μg mL^-1^) and on a mass-to-mass basis PRE is over 1000 times more potent than aciclovir as an antiviral. PRE demonstrated an even greater antiviral response against the aciclovir-resistant strain HSV-ACR with an EC_50_ of 0.016 ± 0.009 μg mL^-1^, whereas aciclovir showed no effect (≥100 μM). Surprisingly neither punicalagin nor ellagic acid elicited any antiviral activity at the concentrations tested (IC_50_ ≥ 100 μM). This result indicates that, unlike in the earlier tests where punicalagin was clearly involved in virucidal activity, the antiviral activity of PRE *cannot* be attributed to this compound. Thus antiviral activity is due to another phytochemical constituent of PRE or, more likely, a combination of constituents. The addition of ZnSO_4_ was not found to increase the anti-replicative potential of the extracts.

**Fig 6:**
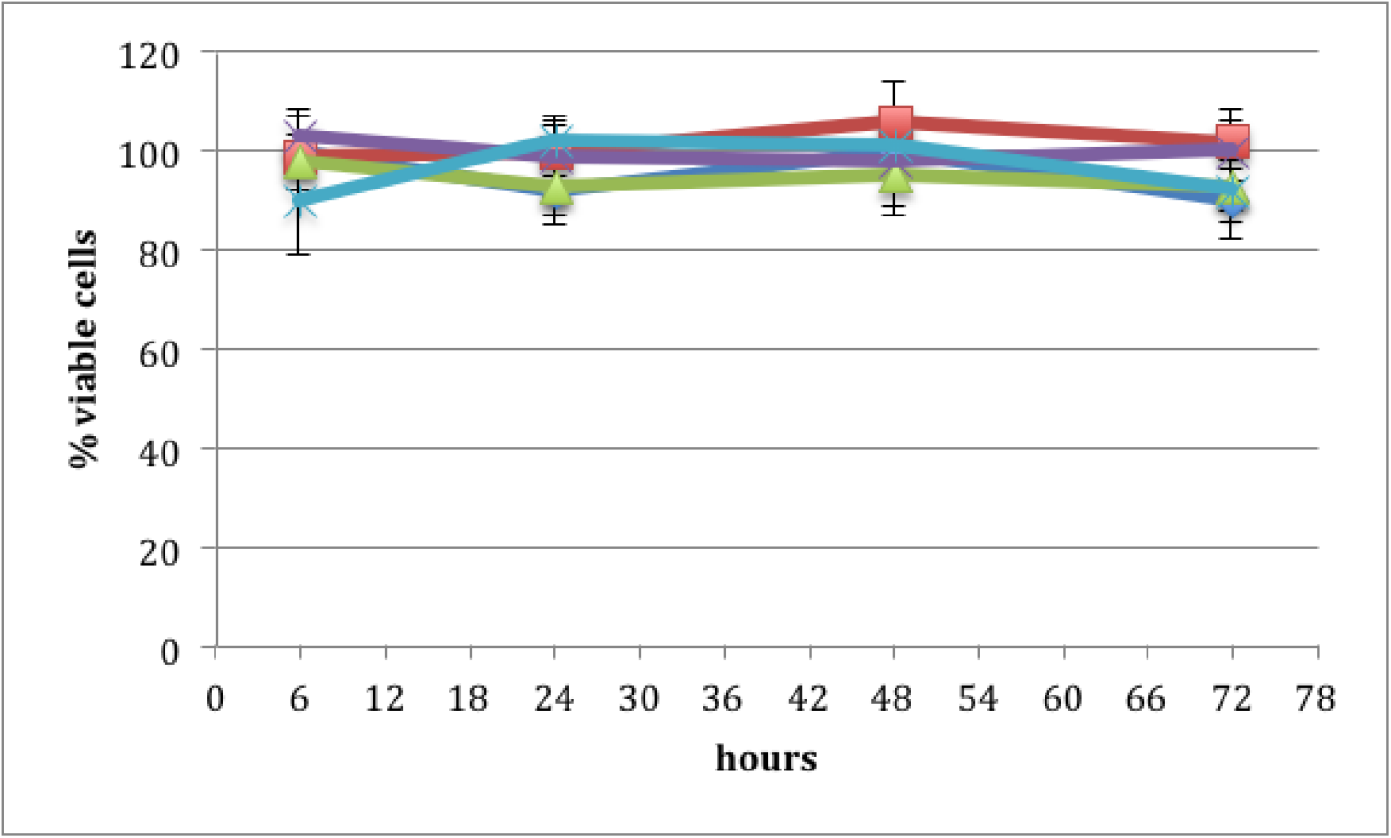
Antiviral EC_50_ curves covering the range 0.001, 0.005, 0.01, 0.5, 1 μg mL^-1^ (n = 3 ± SD).

Antiviral profiles for PRE vs HSV-1, PRE vs HSV-2 ACR, PRE + ZnSO_4_ vs HSV-1 and aciclovir vs HSV-1 are displayed in Fig 6. It is interesting to note the shape of these graphs—the sharp decline in viral activity over a small concentration gradient implies that PRE ± ZnSO_4_ requires a threshold concentration to have any antiviral effect. The shape of the EC_50_ curve for aciclovir exhibits a gradual decline in antiviral activity over a wide concentration range—this and the antiviral activity of against HSV-2 ACR which is resistant to aciclovir suggests that the antiviral activity of PRE is exercised by a different mode of action to that of aciclovir. Synergistic inhibition of ACV-resistant HSV replication was reported using a combination of the ellagitannins: castalagin, vescalagin and grandinin—no mechanism was proposed, although the work did demonstrate the absence of toxic effects [26].

### Cytotoxicity

**Fig 7:**
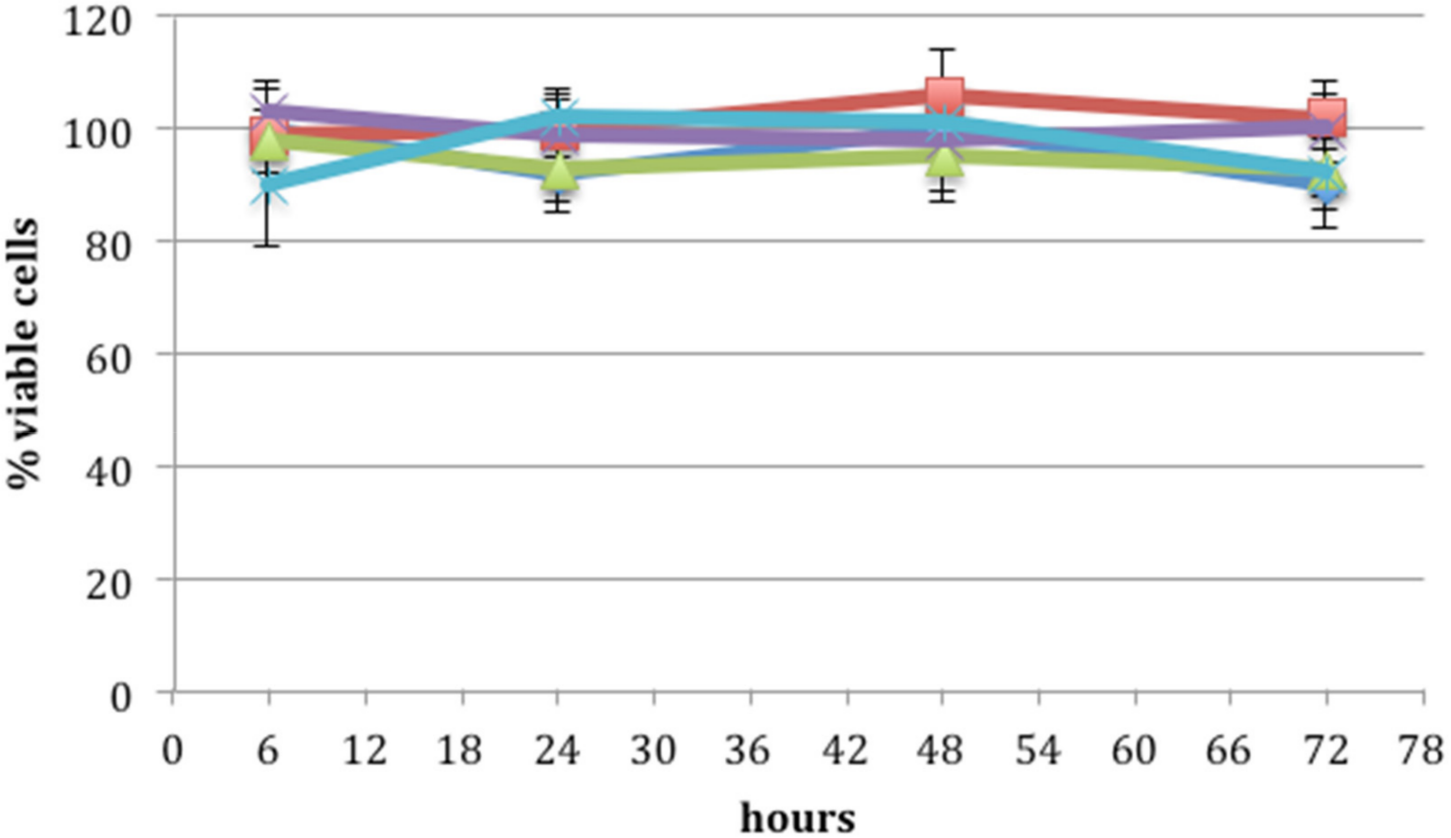
Cell Titre 96 AQueous assay cytotoxic evaluation: the percentage of viable cells remaining after application of total pomegranate tannins (purple), tannin free fraction (light blue), pomegranate rind extract (PRE) 0.1 mg mL^-1^ (dark blue), ZnSO_4_ 0.1 M (red) and PRE 0.1 mg mL^-1^ + ZnSO_4_ 0.1 M (green) after 6, 24, 48 and 72 h (n = 3 ± SD). There was no statistical difference in viable cell count across the duration of the experiments, indicating absence of cytotoxicity with any of the solutions tested.

Cytotoxicity was determined using a widely-used MTS cell proliferation assay. Fig 7 shows the percentage of viable cells after application of PRE 0.1 mg mL^-1^, ZnSO_4_ 0.1 M and phthalate buffer pH 4.5, alone and in combination. Each formulation was made in the phthalate buffer at pH 4.5. There was no significant difference (p >0.05) between the applied formulations at any time point over the 72 h period resulting in no decrease in the percentage of viable cells in comparison to the control. Thus, no cytotoxic effect was observed on the application of PRE and ZnSO_4_ at any concentration used in this work. This is consistent with previous work using a combination of ellagitannins [26]. However, the results for ZnSO_4_ are not in line with earlier work which, based upon the monitoring of DNA synthesis kinetics, predicts cytotoxicity at the levels used in the current work [27]. The Vero (green monkey kidney epithelial) cells (ATCC-CCL81) used in the current work and the African green monkey kidney (AGMK) cell line RC 37 Rita (Italdiagnostics) used by Kumel, are different isolations of the same cell type; both epithelial, so should have similar virucide penetration properties; however, much shorter incubations times were used in the current work.

## General discussion

The mechanism for the potentiated virucidal activity is currently under investigation, with the formation of a Zn-tannin complex being likely involved. Clearly the biphasic concentration-dependant behaviour to ZnSO_4_ of both PRE and punicalagin (Figs 2 and 5) is surprising and counter-intuitive, although each experiment was performed four times. Potentiation increased up to 1.44 mg mL^-1^, after which it decreased before again reaching a maximum, thereafter steadily diminishing. PRE is a complex mixture of phytochemicals and the addition of ZnSO_4_ adds to the complex interplay between constituents, in solution and at the virus surface. Zinc (II) ions are known to be active against HSV [28,29] and limited activity was confirmed in Fig 1. Kumel et al published an in depth study on the inactivation of free HSV by zinc sulphate, and differences may be explained by extended exposure periods in comparison to the current work [27], also the different strains used may have had different sensitivities to zinc [28]. It was shown that punicalagin targeted and inactivated HSV-1 viral particles and could prevent binding, penetration, and cell-to-cell spread [10]. One explanation could therefore be that at higher levels of zinc (which has less potency than PRE+Zn), complexes could form between zinc and other phytochemicals and block the punicalagin/zinc challenge at the HSV surface.

Alternatively, it is feasible that similarities between the virucidal effects of the higher concentrations of ZnSO_4_ combined with PRE and punicalagin can be observed and may indicate that a dual effect is occurring. It is possible that the viral envelope is first compromised by the punicalagin and then bound by the free Zn^2+^ ions, however, at higher concentrations of ZnSO_4_, the Zn^2+^ ions may act first allowing for the punicalagin to then combine with the protein envelope thus destroying it. A similar level of viral destruction is observed within the PRE + ZnSO_4_ system, however, due to the protective nature of a natural product upon its constituent parts, the virucidal activity of punicalagin remains active and thus potentiation is maintained over a greater concentration range of ZnSO_4_ addition (0.14 and 28.75 mg mL^-1^). A decrease in virucidal activity at ZnSO_4_ concentrations 0.29, 0.144 and 0.288 mg mL^-1^ to punicalagin and 2.88 to PRE may indicate competition for a specific protein and or binding site, hindering potentiation. The virucidal mode of action of pomegranate polyphenols has yet to be elucidated, however other research has shown the interaction between polyphenols with envelope viral glycol proteins of influenza [7, 30].

Examination of a range of zinc salts showed that comparable potentiation was achieved to ZnSO_4_ when equal concentrations were employed and the counterion had no adverse effect on the host Vero cells. This is strong evidence that the Zn (II) ion in solution is principally responsible for the potentiating effect. It is interesting to note the comparability of antiviral activity between PRE and the established antiviral aciclovir (Table 2). Even more interesting is the fact that PRE demonstrated an even greater activity against ACR-HSV–the implication is that PRE could have a role in future HSV strategies, when aciclovir approaches the end of its therapeutic lifetime. The addition of ZnSO_4_ was found not to provide enhanced activity and therefore is not involved in a potentiating mechanism during the viral replication stage.

In the current work the Vero host cells were not adversely affected by either PRE or punicalagin, supporting the previous work by Lin et al [10]. Administration of a pomegranate fruit extract, standardized to 30% punicalagins, to Wistar rats showed no adverse effect as high as 600 mg/kg body weight/day [31]. Although a hydroalcoholic pomegranate whole fruit extract at oral doses greater than 70 mg/kg body weight were found to be potentially toxic [32]. In the current work, the extract (with and without ZnSO_4_) was applied locally to the skin and at doses significantly below those used in these earlier papers, further indicating safety for topical use. Indeed, any cytotoxic effects, if they occurred, would be expected to elicit an inflammatory response. In contrast, the anti-inflammatory properties of pomegranate extracts are well documented [33] and our previous work has demonstrated downregulation of COX-2 levels in *ex vivo* skin, following the penetration of PRE and ZnSO_4_ through epidermis and mucous membranes [34,35]. Overall, the absence of cytotoxic effects and demonstrable anti-inflammatory activity suggests the safe application of PRE and zinc ions to skin *in vivo*.

Together with the current antiviral and virucidal data against HSV and aciclovir-resistant HSV, the co-administration of PRE or punicalagin has potential as a novel multi-functional topical therapeutic for coldsores; there is also potential in the treatment of anogenital herpes and viral keratitis. By extrapolation, the topical application of PRE and ZnSO_4_ might be expected to result in virucidal kill outside of the host cell post-budding and help eliminate the viral vesicles. This would have important beneficial consequences for preventing viral transfer by a number of routes, eg human mouth-to-mouth. Additionally, PRE would inhibit viral replication within adjacent cells preventing formation of a viral cluster, which in turn would prevent a cold sore outbreak. Finally, an additional advantage of the proposed therapeutic combination is that the virucidal action involves a potentiated interaction between zinc ions and the complex mixture of phytochemicals, and such potentiation is believed to inhibit microbial resistance by making microbial adaptability very difficult [36,37].
