# Correction: Potentiated virucidal activity of pomegranate rind extract (PRE) and punicalagin against *Herpes simplex* virus (HSV) when co-administered with zinc (II) ions, and antiviral activity of PRE against HSV and aciclovir-resistant HSV

**DOI:** 10.1371/journal.pone.0188609

**Published:** 2017-11-20

**Authors:** David M. J. Houston, Joachim J. Bugert, Stephen P. Denyer, Charles M. Heard

Fig 6 is incorrect. The authors have provided a corrected version of Fig 6 and its caption here.

**Fig 6:**
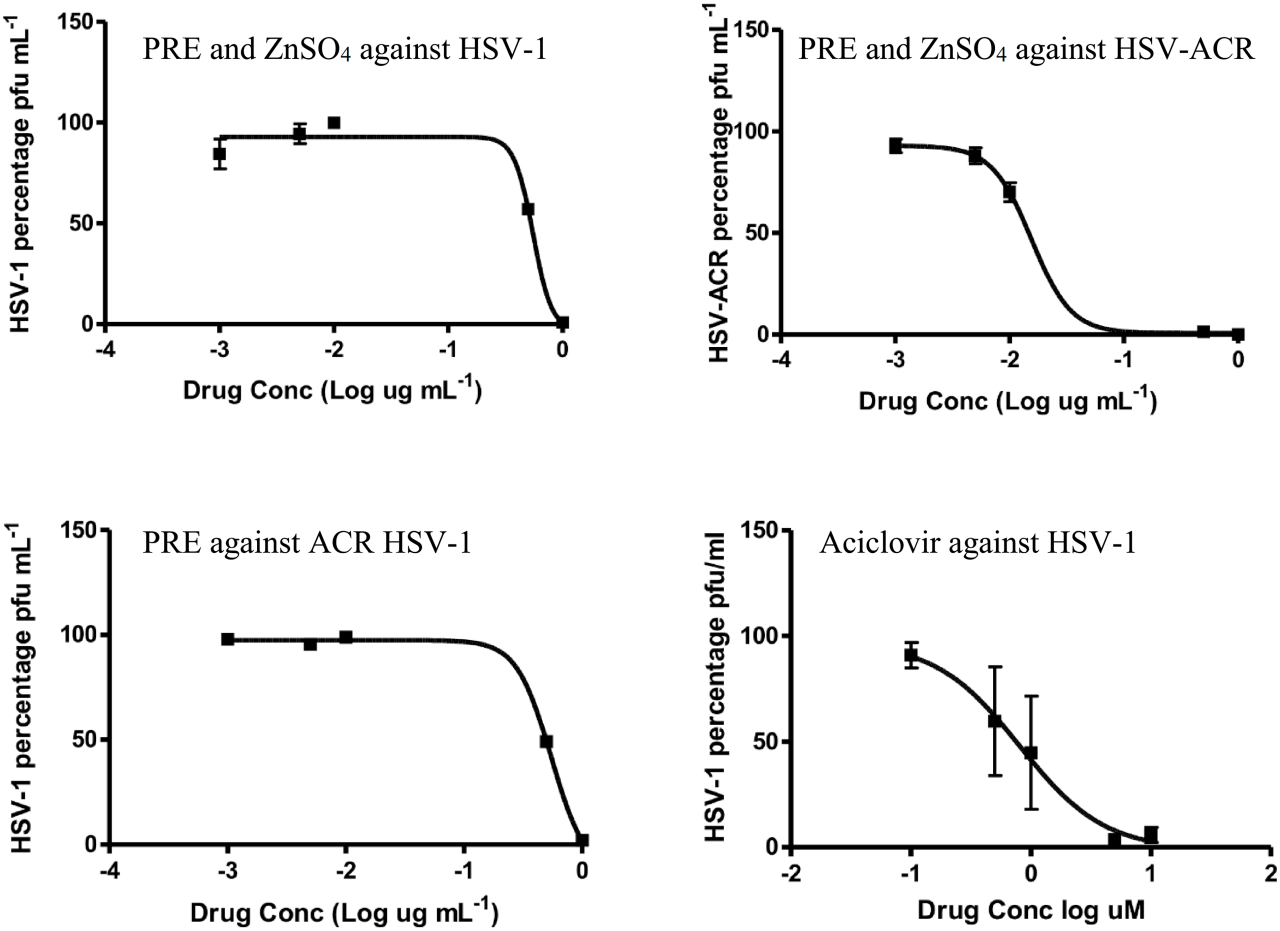
Antiviral EC_50_ curves of Aciclovir (uM), and PRE covering the range 0.001, 0.005, 0.01, 0.5, 1 μg mL^-1^ (n = 3 ± SD).
